# Supplementary material for: Comprehensive Study of Antiretroviral Drug Permeability at the Cervicovaginal Mucosa via an In Vitro Model
Source: Pharmaceutics. 2022 Sep 13;14(9):1938. doi: 10.3390/pharmaceutics14091938 (PMC9504208; doi:10.3390/pharmaceutics14091938)
Supplement: Supplementary file 1 [file pharmaceutics-14-01938-s001.zip › pharmaceutics-1899851-supplementary.pdf]

SUPPLEMENTAL FIGURES

Figure S1

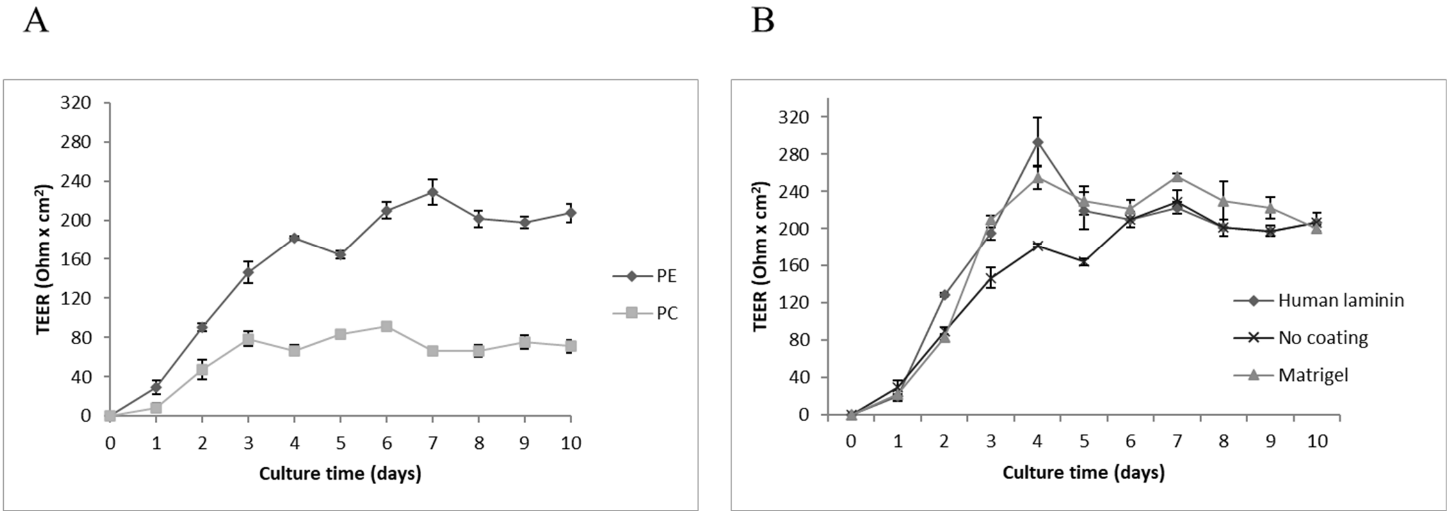

Figure S1. Barrier function of HEC-1A cultures. A) Barrier function on polyester and polycarbonate membranes. Transepithelial Electrical Resistance (TEER) of HEC-1A cell monolayers grown on either polycarbonate (PC) or polyester (PE) membranes was measured with an Epithelial Voltohmmeter. **B)** Effect of laminin or Matrigel coating. Transepithelial Electrical Resistance (TEER) of HEC-1A cell monolayers grown on semi-permeable membranes coated with either human laminin (5 µg/cm^2) or Matrigel®(15 µg/cm^2) is shown in comparison to TEER measured in the absence of coating. Cell density was 2 x 10^5 cells/ Transwell® insert (surface area 1.12 cm^2). TEER values are shown in relation to time.

FIGURE S2

A B

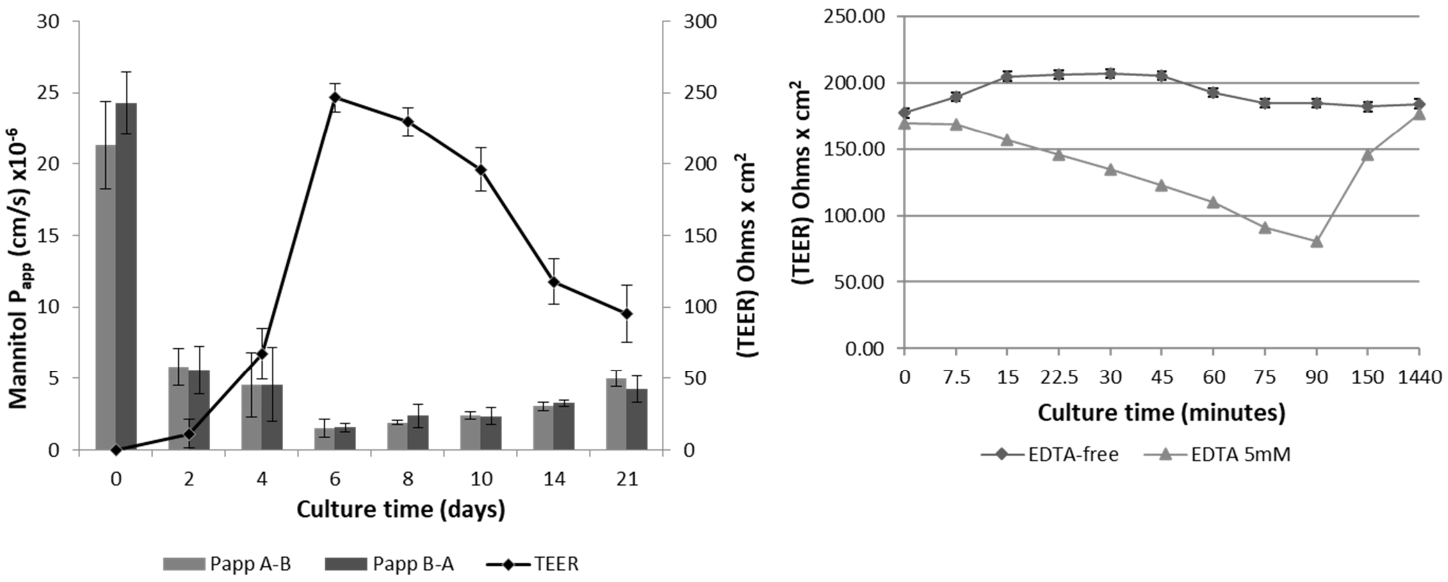

Figure S2. HEC-1A barrier integrity over time and calcium-dependence. A) Barrier integrity assessed by measurement of TEER and by determination of the bi-directional permeability of the para-cellular marker ^{3}H-mannitol. TEER and ^{3}H-mannitol P_{app} values are shown in relation to time. **B**) Effect of EDTA supplementation on barrier integrity. TEER readings over 24 hours in the presence and absence of EDTA.

FIGURE S3

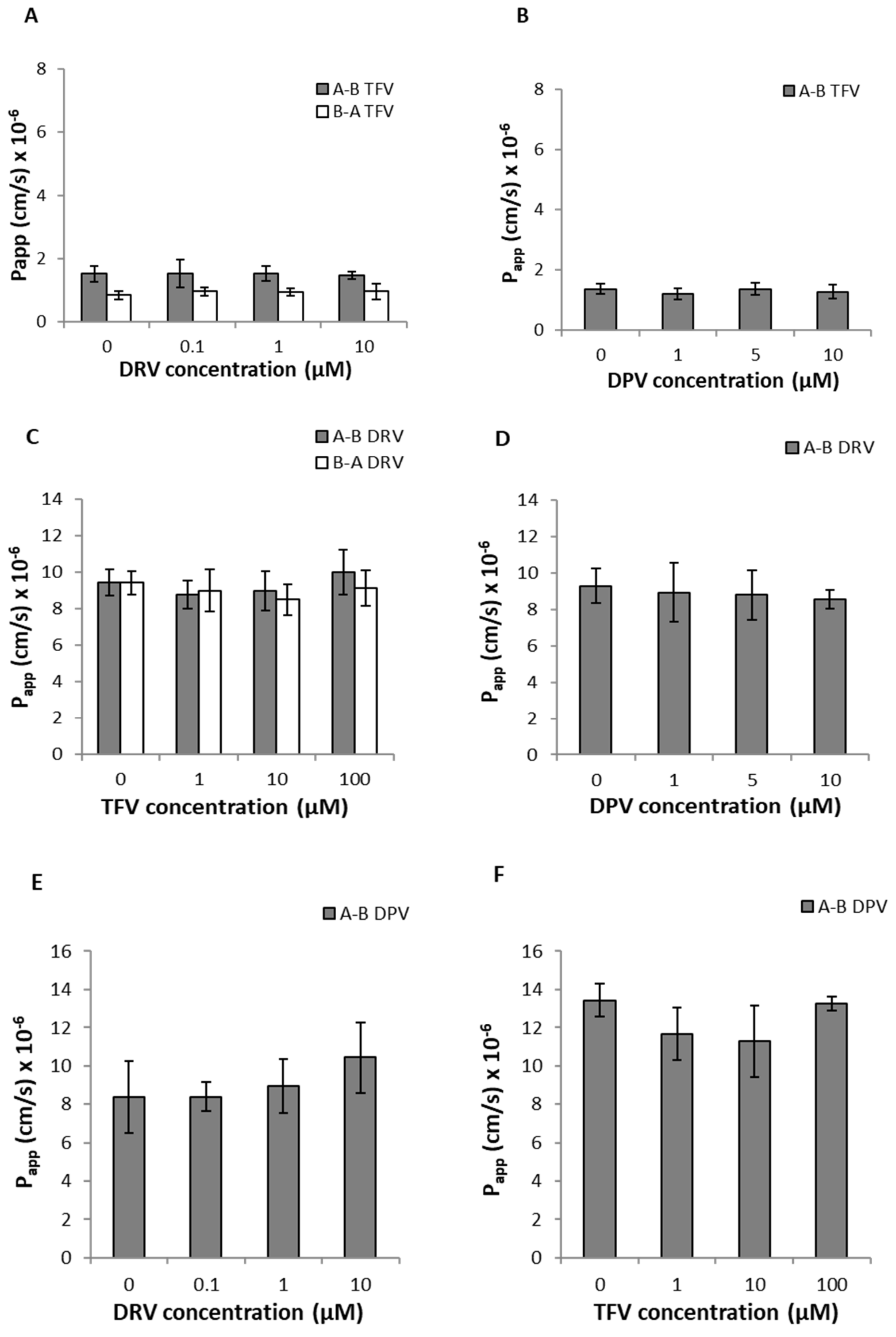

Figure S3. Permeability of ARV drug combinations. Permeability of labelled tenofovir (TFV) (**A-B**), darunavir (DRV) (**C-D**) and dapivirine (DPV) (**E-F**) at 10µM in the presence of either of the other non-labelled drugs at 0-10µM for darunavir and dapivirine and 0-100 µM for tenofovir.

FIGURE S4

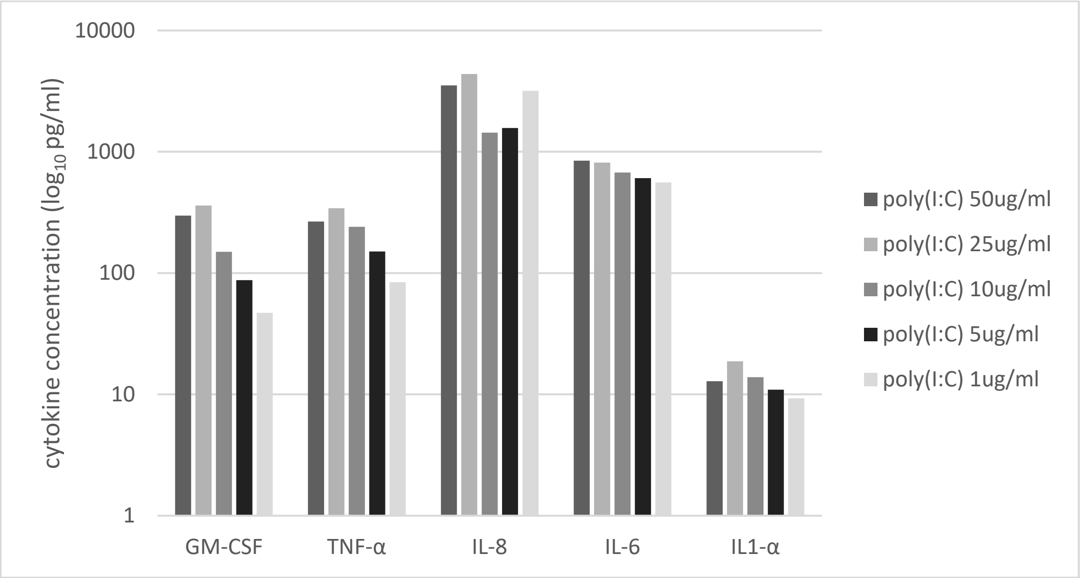

Figure S4. Cytokine production in HEC-1A cells stimulated with poly(I:C). GM-CSF, TNF-α2, IL-8, IL-6 and IL-1α production in response to stimulation with poly:IC at 1-50 µg/ml. Cytokine concentrations (shown in log_{10} pg/ml) were measured using a Magnetic Luminex® Performance Assay kit and analysed using a Luminex® 100 instrument.
